# Modern Transference of Domestic Cooking Skills

**DOI:** 10.3390/nu11040870

**Published:** 2019-04-18

**Authors:** Fiona Lavelle, Tony Benson, Lynsey Hollywood, Dawn Surgenor, Amanda McCloat, Elaine Mooney, Martin Caraher, Moira Dean

**Affiliations:** 1 Institute for Global Food Security, School of Biological Sciences, Queen’s University Belfast, Belfast BT9 5DL, UK; flavelle01@qub.ac.uk (F.L.); t.benson@qub.ac.uk (T.B.); 2 Department of Hospitality and Tourism Management, Ulster Business School, Ulster University, Ulster BT52 1SA, UK; l.hollywood@ulster.ac.uk (L.H.); d.surgenor@ulster.ac.uk (D.S.); 3 Department of Home Economics, St. Angela’s College, F91 C634 Sligo, Ireland; amccloat@stangelas.nuigalway.ie (A.M.); emooney@stangelas.nuigalway.ie (E.M.); 4 Centre for Food Policy, Department of Sociology, School of Arts and Social Sciences, City University London, London EC1R 0JD, UK; M.caraher@city.ac.uk

**Keywords:** cooking, learning, mothers, children, adolescents, obesity, qualitative, environmental influences

## Abstract

As the primary source of learning cooking skills; it is vital to understand what mothers think about the transference of cooking skills to their children. The current analysis aimed to highlight mothers’ perceptions of children’s involvement and cooking practices within the home setting. Sixteen focus group discussions were conducted on the island of Ireland (Republic of Ireland and Northern Ireland [UK]) with 141 mothers aged 20–39 years old. All focus groups were transcribed verbatim and an inductive thematic analysis using NVivo software was undertaken. Seven themes emerged from the dataset; (1) “How we learned to cook”; (2) “Who’s the boss”; (3) “Children in the way”; (4) “Keep kids out”; (5) “Involvement means eating”; (6) “Intentions versus reality”; and (7) “Kids’ ‘interest’ in cooking”. These themes illustrate a lack of cooking skill transference in relation to everyday meal preparation in modern times. The culture of children in the kitchen has vastly changed; and opportunities for children to learn basic skills are currently limited. Further research is required to confirm the findings that emerged from this analysis.

## 1. Introduction

The possession and application of cooking skills can have numerous health benefits including a greater diet quality, weight control, and even longevity of life [1,2,3,4]. In light of this, there has been a resurgence in cooking skills education and a push for the re-skilling of the general population to reinvigorate meal preparation in the home environment [5,6,7]. Cooking skills interventions are also being increasingly implemented as childhood obesity prevention strategies or as essential components in multidisciplinary prevention approaches [8,9,10] as recommended by the World Health Organization [11]. These interventions are utilized as a means of enabling children and adolescents to prepare healthy meals as alternatives to the use of ready meals and the consumption of food outside the home [2,4,11,12]. The need for children and adolescents to learn these cooking skills has been highlighted as an important life skill [11]. Research suggests that adolescents who are involved in home meal preparation present with a higher diet quality than their non-food preparing counterparts [13]. In addition, learning cooking skills at younger ages has also been linked to skill maintenance through to adulthood, cooking confidence and a better diet quality [14]. 

Despite understanding the importance of cooking skills development at a young age, the optimal source for learning these skills has been debated. While a substantial emphasis has been placed on teachers through the education system [14,15,16,17], other sources are also important. The mother has been consistently identified as the primary source for learning cooking skills [14,18]. In addition, mothers have been identified as key influencers of children’s weight status [19,20,21] and learning from the mother is associated with greater cooking confidence and less consumption of unhealthy foods, emphasizing the valuable role of the mother as a primary source of learning [14]. However, due to the increasing demand of current modern lifestyles and external pressures, some research suggests that mothers may no longer possess the necessary skills or time to prepare a healthy diet [22] and, therefore, may no longer be able to pass on cooking skills to their children. Anecdotally, it has been suggested that there is a lack of skill transference occurring from mothers to children, however, this phenomenon has not been explored qualitatively in order to understand why this may be happening. Thus, the role of the mother as a current source of learning must be examined. 

Given their key role as influencers on children’s weight status and the primary source for learning skills that enable the preparation of a healthy diet, it is vital to understand what mothers think about the importance of cooking skills development in their children and how this can be best modeled in the home setting to promote confidence and learning and the passing on of these skills to their children. A greater understanding of mothers’ attitudes, behaviors and feelings could help to inform strategies to promote and encourage the learning of cooking skills. Therefore, this analysis aimed to highlight mothers’ perceptions of children’s involvement and cooking practices within the home setting.

## 2. Materials and Methods

### 2.1. Focus Group Recruitment

The participants were recruited to partake in a cooking from “scratch” experiment [23], with an immediate follow up focus group, in a room adjacent to the kitchens, to discuss their perceptions of the cooking experiment and experiences of cooking in general. They were recruited by Social Market Research (a market research company based in the United Kingdom and Republic of Ireland (ROI)). Overall, 160 mothers (20–39 years old), both employed and unemployed, who were responsible for the main meal preparation at least 3 times per week, and who had at least one child (under the age of 16 years) currently living in their household, with a range of children’s ages, were recruited, with the final sample consisting of 141 (due to non-attendance). Participants could also not have any strict dietary requirements (such as lactose intolerant or vegetarian, for the purposes of partaking in the cooking experiment). Sixteen focus groups were conducted, with eight held in Sligo, ROI and eight held in Coleraine, Northern Ireland (NI). Each group had six to ten participants. Participants were recruited from a 30-mile radius of both sites and included both urban and rural participants. Results are treated as one island of Ireland sample. Sociodemographic and food-related characteristics were collected before the beginning of the cooking experiment and focus groups. 

### 2.2. Focus Group Procedures

The focus groups were conducted in line with the principles outlined in Kreuger and Casey [24]. The discussions were facilitated by an experienced moderator (DS) and an assistant moderator (FL). The focus groups followed a guided open-ended questioning route relating to experiences of the cooking experiment and cooking habits and behaviors in general, an outline of the topic guide can be seen in Table 1. Probing questions relating to children’s involvement in cooking were used for all mentions of children. The focus group topic guide was developed from a literature review [25] and earlier individual interviews conducted on the island of Ireland relating to cooking behaviors and habits [26]. Additionally, the guide was piloted with two focus groups, one group who had not conducted the cooking experiment (for general flow of the question route and the wording of the questions) and one group after piloting of the cooking experiment. The moderator emphasized the importance of all participants contributing to the discussion, and that all opinions and points were equally valid. The assistant moderator was present to take notes and facilitate the direction and flow of the discussion. All participants were assured of their confidentiality and all discussions were audio recorded. Each focus group discussion lasted between 50 and 65 min. Upon completion of the focus group, each participant was thanked and given an honorarium (£50/€50) and a cookbook to compensate for their time (including the cooking experiment) and travel. 

### 2.3. Institutional Review Board

The study was conducted in line with the guidelines laid down in the declaration of Helsinki. All participants provided written and verbal consent and were aware that they could withdraw from the research study at any point. The study was approved by the Research Ethics Committee within The School of Biological Sciences at Queen’s University Belfast. 

### 2.4. Analysis of Focus Group Transcripts

Focus group discussions were professionally, independently transcribed verbatim and checked for accuracy by the moderator and then imported into Nvivo 11 (QSR International Pty Ltd, Doncaster, Victoria, Australia) for analysis. An inductive thematic analysis in line with Braun and Clarke [27,28] was undertaken. The dataset used in this analysis involved all instances where participants discussed children in the kitchen environment including discussion of when they were children.

All transcripts were read and re-read by two of the authors in order to achieve data immersion. Subsequently, all data relating to “children” were coded for this analysis. The next phases involved grouping codes together to form potential themes, inspecting these themes for overlap and where necessary refining the themes. This refinement ensured that there were “clear and identifiable distinctions” between the themes and that there was no overlap [27]. 

An inter-rater process was used throughout the entirety of the analysis. Initially, two researchers (FL, a sport and health scientist, and TB, a health psychologist) independently coded 3 randomly selected transcripts (18% of the transcripts). The coders had an initial agreement of 90% on the coding of the transcripts. The codes were discussed to verify their applicability to the data, and agreement was reached on all codes upon discussion. Following this, FL coded the remaining transcripts and TB coded a further 5 transcripts, leading to 97% coding agreement across these 8 transcripts. Then, FL grouped codes to form potential themes. The themes were inspected for overlap and consensus was reached on all themes through discussion (TB and MD, a consumer food choice psychologist). Illustrative quotes were then extracted from the data to demonstrate typical views within each theme. Data saturation was reached within this topic area as no new codes appeared after the first ten transcripts. Sociodemographic data was summarized using SPSS v22 (IBM Corporation, Armonk, NY, USA, 2013).

## 3. Results

### 3.1. Participant Characteristics

The characteristics of the 141 participants can be seen in Table 2. Mean age was 30.45 years (SD 5.70).

Mothers with a wide range of food related behaviors and levels of cooking and food skills confidence were recruited, as shown in Table 3.

### 3.2. Overview of Themes

Through thematic analysis of the focus group transcripts, seven themes were constructed: (1) “How we learned to cook”; (2) “Who’s the boss?”; (3) “Children in the way”; (4) “Keep kids out”; (5) “Involvement means eating”; (6) “Intentions versus reality”; and (7) “Kids’ interest”. 

The culture of children being present in the kitchen appeared to revolve around the mother–child dynamic, with an underlying shift in this dynamic, from mothers being in control in previous generations to currently the child dictating meal choice. This shift appeared in two themes, “How we learned to cook” and “Who’s the boss?”, as described below.

(1) **How we learned to cook.** Here, participants discussed how they were present in the kitchen from a young age and how they learnt to cook from their mothers.

“*When I was younger mum always had us in the kitchen and teaching us how to cook and that.*”

ROI FG2

The majority of participants claimed that they had to help with the dinner. There was no choice and it was what was expected of them and part of their family dynamics.

“*When we were younger my mum was out working an awful lot and I was minding the younger ones… I just remember cooking at a really young age...*”

“*Oh yeah I’d have done that stand on the chair [because] you couldn’t reach.*”

ROI FG6

Some of the mothers noted that in some instances they had to assume the role as meal preparer in its entirety for their families. This is a sharp contrast to current practices where only two mothers of the 141 involved in the focus groups mentioned their older children helping with every day meal preparation. Thus, the culture of children helping in the kitchen and “doing jobs” in the kitchen is a rarity.

(2) **Who’s the boss?** This theme highlights how the power of food choice has shifted to the child. This shift has put extra pressure and stress on the mother and has changed the ‘cooking a meal’ experience as well as what preparing a meal actually entails. Mothers claim that children dictate what type of food is prepared by being fussy eaters or liking different textures or by how food is eaten (for example with their hands).

“*We all have different dinners in our house. So, because my wee (small/young) boy is like three so he’s like ‘I don’t like that, I don’t like that’ and my wee girl she would rather sit with like salads, like she just loves all that so it’s really difficult in my house….My wee girl hates mince so like, you know your spag bol (spaghetti Bolognese) that you would love really quick or like cottage pie or anything like that she’s ‘no’ so I know she’s not going to eat it… There’s always one with something different in my house.*”

NI FG5

In every focus group, it was mentioned that the participants cook to cater to their children’s wants and needs, *“What the wee ones [the children] want to eat”* (NI, FG 8). Sometimes they are unaware of this level of control, and believe that they are making “compromises”, although still preparing what the child wants.

“*[There] might be something that you want to cook but you know they’re not going to eat it so you just have [to] compromise and go with what you know is going to be eaten as well.*”

ROI FG6

If mothers do not want to eat what their children are eating, they make multiple dinners to avoid arguments, tantrums or revolt. To cope with the demand of having to make multiple dishes, some participants resorted to the use of convenience products instead of deciding what is to be eaten.

From these added stresses and pressures in the kitchen, two negative themes arose, “Children in the way” and “Keep kids out” with over three quarters of the focus groups having discussed children being in the way of their cooking and not wanting the children in the kitchen when they are cooking.

(3) **Children in the way.** This strongly presented theme revolves around the impact of having children present in the kitchen on the mother’s current cooking practices. It highlights how children, “*little tigers*”, are in the way when mothers are trying to prepare a meal, hanging on to the mothers or shouting and pulling at them.

“*I have 2 kids running about pulling me grabbing me mummy, mummy, mummy…then babies looking fed and it’s just madness.*”

NI FG2

Some of the mothers mentioned that they would like a “*babysitter*” to occupy the children so that the participants were able to cook a meal. When children have disabilities requiring extra time or can have problems with food, this results in less time for the mother to cook. When faced with these situations, mothers tend to cope by cooking quickly, and by taking shortcuts, such as using convenience products, or by cooking food when the children are out of the way in school or in another room playing.

“*Like I have 5 kids fluttering around and you just don’t have time to be standing with a flipping wooden spoon…*”

ROI FG1

(4) Keep kids out. This theme revolves around what stops mothers having their kids in the kitchen and involving them in the cooking process. The participants stated that they are too busy to deal with the mess children create in the kitchen as at times children can leave the kitchen looking like a *“bomb site”.*

“*See because they’ll just create such a mess.*”

NI FG8

Along with the potential mess of letting the children in the kitchen, participants also noted concerns over the safety of having children in the kitchen.

“*Do you not be worried about them using a baker [oven] and things at that age?*”

“*Well I don’t let my 4 year old use the cooker.*”

“*Oh no obviously.*”

“*The 13 year old she says yeah they have to use the cooker in school and stuff so.*”

“*Oh I see now aye it just sounds really young still doesn’t it though.*”

NI FG4

While not as prevalent, there were also positive themes relating to the transference of skills from the mother to the child—“Involvement means eating” and “Intentions versus reality”. The themes were mentioned multiple times within a smaller number of focus group discussions. While some transference of skills to the child occurring may happen as a result of these themes, the skill level being transferred may not occur frequently, not happen in all situations, and the type of skills transferred may not be optimal for everyday living.

(5) Involvement means eating. The “involvement means eating and trying foods” theme highlights the participants’ perceptions about their children eating different types of food or food they have previously refused when they are involved in the preparation of the food.

“*My wee girl doesn’t like vegetables or anything I think 2 or 3 weeks ago I got her to help me make lasagne, she loves pasta. I got her to help me make it and oh my god there was a clear plate so she loved it, she doesn’t like peas or carrots or courgettes (zucchinis) or onions or anything like that everything in and then she ate it all, no questions asked.*”

NI FG5

Some of the participants felt that children experiencing the food ingredients in the kitchen rather than it being presented to them as a complete dish to eat removes the fear of the unknown.

“*Whereas if she was actually helping to make (dinner)… She’ll eat anything if she kind of knows what it is but whenever they don’t seem to know what it is they are just a bit wary of it.*”

NI FG3

(6) **Intentions versus reality.** This theme addresses the concept of cooking with children. Mothers in general expressed a desire for their children to be able to cook to help them in their future.

“*Yeah and you want them to be able to know how to cook, so that when they hit 18 they wouldn’t just live on little packets of you know pasta or takeaway, that they have some idea, they can come in they can make a Spaghetti Bolognese or they can do the basics.*”

ROI FG1

However, when mothers discuss instances of when they cook with their children they mention baking, which is seen as a fun activity or random dishes that children pick to cook rather than everyday dishes that form their diet.

“*My little one loves to bake and stir and she’ll make pizza for her friends, you know, she loves doing stuff like that and all sorts of nonsense.*”

NI FG8

Participants also referred to the occasional instances where they had the children make the meal with them, to try and encourage them to eat the food, as discussed in the previous theme. In addition, the participants mentioned eating food they do not like that children have prepared to encourage the children to cook. 

In the final theme, participants discussed their child’s interest or stated that they were not interested in cooking. It was unclear whether children showed an interest in cooking due to greater cooking exposure or whether children were naturally interested. This theme is detailed below.

(7) **Kids’ “interest” in cooking.** This theme was present in eight of the 16 focus groups, however, not all instances were positive, as some reported that their children had no interest in cooking.

“*(Cooking) bores my 10 year old, (they’re) not interested.*”

NI FG4

Some mothers commented on their children’s interest in cooking and that they wanted to cook and learn to cook at home or in home economics.

“*Well he’s doing home economics and all in school and he loves to cook yeah.*”

ROI FG5

Some participants proposed that the child’s interest in cooking arises as a way of gaining a sense of independence instead of just having their ‘*dinner set down in front of them*’ and that they gained a sense of pride and achievement.

## 4. Discussion

This analysis investigated the phenomenon of transgenerational cooking skills transference in modern times. The findings indicate that the culture of children being in the kitchen has vastly changed, and opportunities for children to learn basic and fundamental food related skills are not present in the current climate. Recently, home economics teachers in Australia highlighted how children “don’t have a clue” about food skills when they enter schools due to parents’ time constraints and perhaps due to parents’ limited skills [30]. They stressed the importance of adolescents learning cooking and food skills to enable them to make informed food choices [30].

In line with recent research showing that the mother is the primary source of learning [14,18], the transference of cooking skills from the mothers’ mothers was found. This relates to past behaviors and learning and is therefore logical that these participants report the same source that has been discussed previously in the literature. Mothers reported how they were involved in the cooking process in the kitchen and were sometimes responsible for cooking when they were children. This is in contrast to current practices, with a minority of mothers mentioning their children helping with the everyday meal preparation. The mothers’ felt a lack of control when the children were in the kitchen and the children distract the mothers from cooking. This caused extra stress and negativity to the whole cooking experience. In addition, mothers did not want their children in the kitchen because of safety concerns, not considering that they themselves had previously been in the kitchen at a similar young age. Additionally, although there was a mention of children using different appliances at certain ages in school, some participants still showed a hesitancy about this and there appeared to be uncertainty over the appropriate age for including children in cooking. Furthermore, the participants did not want to have to clean up the mess created by having children in the kitchen. This idea of having to clean up after the children may be a misunderstanding because, as part of learning basic food skills, children need to learn about cleaning up after cooking and about learning to cook in a neat and safe manner [31]. However, this may also reflect a societal change, where more women are in employment and may have limited time to undertake household responsibilities [32] and, therefore, removing children from the kitchen may be seen as a means to reduce their workload. However, the removal of the children from the kitchen may result in a lack of skill transference.

Furthermore, children are currently dominating meal choice, which in turn influences the type of cooking that occurs and increases the pressure on mothers through the cooking of multiple meals. This is in direct contrast to Lai-Yeung [33] who found that Hong Kong mothers dictate food choice decisions. However, this theme is in line with other western studies [26,34,35] where there has been an emergence of the “junior consumer” deciding on the food to be purchased and prepared, suggesting the existence of cross-cultural differences in this area. 

The “rareness” of skill transference occurring from parent to child was alluded to by Lai-Yeung [33] and minimal transference may have been occurring from a minority of participants to their children. Cooking experiences mentioned tended to be fun activities, not daily occurrences and not daily meal preparation. Although any level or element of cooking is positive, the infrequency and type of cooking contribute to the lack of transference of skills. 

These changes in cooking practices may have detrimental effects on the learning of cooking skills, where children do not perform the everyday tasks in meal preparation (including food skills such as cleaning up safely after creating a mess in the kitchen), or are being removed from the kitchen and in turn are missing crucial opportunities to learn basic, fundamental skills. This may be contributing to the disappearance of important life skills. 

Involving children in cooking has been used previously as a successful strategy to overcome picky eating [36], increase consumption of healthier foods [37] and to increase willingness to taste unfamiliar foods [38]. This strategy has the potential to combat the lack of transference of skills. In addition, it may help to reduce the impact of the child dictating the meal choice, as the child would be more open to and aware of different foods, potentially increasing their food neophilia (i.e., their openness/willingness to trying new and novel foods). Greater willingness to try new foods could be promoted as a key benefit in learning cooking skills.

Not everyone is interested in cooking; however, as it is a valued life skill, it would be appropriate to encourage it across all genders. Parents have been previously found to be supportive of this skill transference [33]. Mothers in this research felt that cooking could be a way for their children to assert their independence and achieve a sense of pride. The ability for cooking skills to be empowering in adult settings has been proposed in previous research [22] and from our results it is suggested that the sense of achievement and empowering element of having cooking skills is not only found in adults but across all ages. Teaching children skills in other domains such as in research has been shown to empower children to become engaged researchers and that the greater is the participation, the more experienced and competent the child becomes, and the greater is the sense of empowerment as the child becomes more effective in the execution of skills [39]. How cooking skills can provide a sense of independence and pride could be a key focus for the promotion of cooking skills in combination with health benefits.

### 4.1. Limitations

A limitation to this research is that the sample consisted only of mothers. However, mothers have been reported as the primary source of learning cooking skills [14] and an investigation into the current situation was therefore warranted. As some of the participants were in employment, further research is needed into the role of the primary daytime carer of the child, for example grandparents, in the transference of cooking skills. Additionally, there may be some cross-cultural differences in the findings. While there may be limited generalizability (as is inherent in qualitative research), the large number of focus groups with a broad range of mothers with varying food-related behaviors and practices allowed for an extensive range of descriptive ideas that may contribute towards reducing these differences. Further quantitative research in this area could help with the generalizability of the results.

### 4.2. Implications for Research and Practice

From the above findings, the authors propose two key recommendations: (1) upskilling of mothers’ food skills in relation to organizing meals and preparation time, to allow for children to be involved and to reduce stress; and 2) creating an awareness of the importance of kids being in the kitchen and helping with everyday meal preparation. These recommendations may have implications for future interventions and future research. Recommendations 1 and 2 could be implemented through increased numbers of family inclusive interventions, to help parents to acclimatize to children cooking alongside them. This may show parents that children can be involved and assist with everyday meal preparation and highlight different age appropriate cooking skills and tasks. Our findings support the idea of having interventions that focus on the mother–child dyad. Inclusive family interventions may promote the use of these cooking skills to prepare healthy and nutritious meals. 

The inclusion of practical cooking skills in any nutrition component of an obesity prevention program is key, to provide individuals with the necessary skills to prepare healthy food [22]. Future longitudinal research could investigate the use of cooking skills over the life course and its impact on weight status. Additionally, the influence of parenting styles on the learning of cooking skills is a key novel area that requires further investigation, as some of the findings in this analysis show children dictating food choices instead of parents which could suggest a shift from the more authoritarian/authoritative parenting approaches in the past to a more permissive/uninvolved style [40]. This could be problematic as a recent review of parenting styles and future weight status [41] suggests that an authoritative parenting style may have a protective role against future overweight and obesity [41]. Although mothers currently have the responsibility of passing on cooking skills, with shifting family dynamics and the increase in the “stay at home husband”, future research could investigate if fathers are currently involved in meal preparation and whether this impacts on children’s involvement in meal preparation.

Previous research has stressed the importance of cooking skills education through the educational system [14,15,16,17], however, initially the role played by the educational system was to expand upon the skills learned in the home environment [17]. Presently, the lack of skills that children present with at school has been highlighted [31] and a push for compulsory practical education can be seen in numerous countries [14,15,31]. It is suggested that a combination of the above methods is essential to promote the use of cooking skills and to empower individuals to prepare healthy nutritious meals to improve their diet quality as a strategy for obesity and other diet-related disease prevention and management. 

## 5. Conclusions

The findings suggest that the culture of children cooking in the kitchen has vastly changed, and opportunities for children to learn basic and fundamental skills are currently lacking which may have detrimental effects on their diet quality. The qualitative nature of the study provides insights into why mothers may not involve their children in cooking including children creating a mess and distracting the mothers from their own cooking and may help with the design of future interventions targeting these behaviors. In addition, a greater awareness of age-related skills and tasks for children in the kitchen should be promoted. 

## Figures and Tables

**Table 1:** Outline of the focus group topic guide.

Topic	Description/Question
Introduction	Facilitator introduction Boundaries of the focus group and contracting including recording consent
Confidence Levels	What was your perceived confidence ability in cooking lasagne from scratch prior to the task? Has this confidence changed? How?
Barriers/facilitators to cooking from scratch	How challenging did you find the task? What were the most/least challenging aspects? What would encourage/discourage you to cook using fresh ingredients at home? What additional barriers do you consider prevent you from cooking in the home environment?
Identification of skills used	What skills can you identify in cooking lasagne? Do you consider these skills achievable in your home? Which skills did you consider most challenging? Would you practise these to enable you to cook this or a similar dish at home?
Use of Technology	Do you have home access to the internet? Do you use the internet to assist with learning practical skills? Can you think of an example? Would you consider using technology to assist with home cooking? What part do you consider technology can play in promoting cooking from scratch in your own homes?
Transferability of skills/learning to the home setting	Considering the skills you identified earlier—can you think of other meals where you might incorporate skills developed today, or different ingredients, for example, you may like to change or incorporate ingredients to make the dish healthier or more preferable for the family’s taste?
Summary and ending	What would you do differently next time? Do you have anything else you would like to add or feel we have missed? Thank you and close

**Table 2:** Demographic and Sociodemographic Characteristics of Focus Group Participants (*N* = 141).

Characteristic	*N* = 141	
	*N*	%
Country of Residence		
Northern Ireland (NI)	77	54.6
Republic of Ireland (ROI)	64	45.4
Highest Level of Education		
None/Primary School	4	2.8
Junior cert/GCSE *	17	12.1
Leaving cert/A level **	18	12.8
Additional training	67	47.5
Undergraduate University Degree	24	17.0
Postgraduate University Degree	9	6.4
Number of Children in household under 16		
1	69	48.9
2	43	30.5
3	21	14.9
4+	6	4.2
Perceptions of their weight status		
Very underweight	1	0.7
Slightly underweight	5	3.5
About the right weight	51	36.2
Slightly overweight	57	40.4
Very overweight	26	18.4
Follow a special diet		
No	99	70.2
Yes—self determined	39	27.7
Yes—prescribed by medical professional	3	2.1
Work or has worked in the “food or hospitality industry” preparing food		
Yes	50	35.5
No	91	64.5

* Junior Cert (ROI)/GCSE (NI)—Age 15/16 years, exams taken midway through secondary school. ** Leaving Cert (ROI)/A level—Age 17/18 years, final exams taken in secondary school.

**Table 3:** Food Related Characteristics of Participants (*N* = 141).

Food Related Variables	Number		%	
	141		100	
Typical ingredients used in meal preparation				
Mostly pre-prepared ingredients and assemble	5		3.5	
Mostly pre-prepared ingredients, some fresh, basic or raw ingredients	68		48.2	
Mostly fresh, basic or raw ingredients, some pre-prepared ingredients	59		41.8	
Only fresh, basic or raw ingredients	9		6.4	
Consumption of meals prepared outside the home (e.g. restaurant takeaway food, Chinese, fish and chips, etc.)				
Everyday	6		4.3	
4–6 times per week	6		4.3	
2–3 times per week	7		5.0	
Once per week	62		44.0	
Less than once per week	54		38.3	
Never	6		4.3	
*Breakfast (*N* = 138)				
Never	8		5.8	
Weekdays	16		11.6	
Weekends	18		13.0	
Both	96		69.6	
Lunch (*N* = 137)				
Never	4		2.9	
Weekdays	25		18.2	
Weekends	9		6.6	
Both	99		72.3	
Dinner (*N* = 136)				
†Never	1		0.7	
Weekdays	13		9.6	
Weekends	3		2.2	
Both	119		87.5	
	Minimum	Maximum	Mean	SD
‡Cooking Skills Confidence (*N* = 139)	30	97	65.74	14.66
Food Skills Confidence (*N* = 140)	14	124	84.15	20.41
Cooking Identity (*N* = 138)	7	30	16.67	3.82
Food Neophilia (*N* = 140)	3	14	6.33	2.28

* Participants were asked “Do you prepare/cook Breakfast/Lunch/Dinner during weekdays, weekends or both? (including preparing cold dishes like salads, or reheating ready-made foods)” † This participant was responsible for the meal preparation of their household, however, this consisted of collecting the takeaway. ‡ Cooking and food skills confidence were measured using a paper pen version of the validated measure in Lavelle et al. [29].

## References

[1] McGowan L., Pot G.K., Stephen A.M., Lavelle F., Spence M., Raats M., Hollywood L., McDowell D., McCloat A., Mooney E. (2016). The influence of socio-demographic, psychological and knowledge-related variables alongside perceived cooking and food skills abilities in the prediction of diet quality in adults: A nationally representative cross-sectional study. Int. J. Behav. Nutr. Phys. Act..

[2] Wolfson J.A., Bleich S.N. (2015). Is cooking at home associated with better diet quality or weight-loss intention?. Public Health Nutr..

[3] Chen R.C.Y., Lee M.S., Chang Y.H., Wahlqvist M.L. (2012). Cooking frequency may enhance survival in Taiwanese elderly. Public Health Nutr..

[4] Van der Horst K., Brunner T.A., Siegrist M. (2011). Ready-meal consumption: Associations with weight status and cooking skills. Public Health Nutr..

[5] U.S. Department of Health and Human Services, National Institutes of Health, National Cancer Institute (2016). Down Home Healthy Cooking. http://www.cancer.gov/about-cancer/causes-prevention/risk/diet/down-home-healthy-cooking.pdf.

[6] Reicks M., Trofholz A.C., Stang J.S., Laska M.N. (2014). Impact of cooking and home food preparation interventions among adults: Outcomes and implications for future programs. J. Nutr. Educ. Behav..

[7] Jones M., Dailami N., Weitkamp E., Salmon D., Kimberlee R., Morley A., Orme J. (2012). Food sustainability education as a route to healthier eating: Evaluation of a multi-component school programme in English primary schools. Health Educ. Res..

[8] Davis J.N., Ventura E.E., Cook L.T., Gyllenhammer L.E., Gatto N.M. (2011). LA Sprouts: A gardening, nutrition, and cooking intervention for Latino youth improves diet and reduces obesity. J. Am. Diet. Assoc..

[9] Morgan P.J., Collins C.E., Plotnikoff R.C., Callister R., Burrows T., Fletcher R., Okely A.D., Young M.D., Miller A., Lloyd A.B. (2014). The ‘Healthy Dads, Healthy Kids’ community randomized controlled trial: A community-based healthy lifestyle program for fathers and their children. Prev. Med..

[10] Isoldi K.K., Calderon O., Dolar V. (2014). Cooking up energy: Response to a youth-focused afterschool cooking and nutrition education program. Top. Clin. Nutr..

[11] World Health Organisation (2016). Report of the Commission on Ending Childhood Obesity. http://apps.who.int/iris/bitstream/10665/204176/1/9789241510066_eng.pdf?ua=1&ua=1.

[12] Nelson S.A., Corbin M.A., Nickols-Richardson S.M. (2013). A call for culinary skills education in childhood obesity-prevention interventions: Current status and peer influences. J. Acad. Nutr. Diet..

[13] Larson N.I., Perry C.L., Story M., Neumark-Sztainer D. (2006). Food preparation by young adults is associated with better diet quality. J. Am. Diet. Assoc..

[14] Lavelle F., Spence M., Hollywood L., McGowan L., Surgenor D., McCloat A., Mooney E., Caraher M., Raats M., Dean M. (2016). Learning cooking skills at different ages: A cross-sectional study. Int. J. Behav. Nutr. Phys. Act..

[15] Wolfson J.A., Frattaroli S., Bleich S.N., Smith K.C., Teret S.P. (2017). Perspectives on learning to cook and public support for cooking education policies in the United States: A mixed methods study. Appetite.

[16] Hartmann C., Dohle S., Siegrist M. (2013). Importance of cooking skills for balanced food choices. Appetite.

[17] Caraher M., Lang T. (1999). Can’t cook, won’t cook: A review of cooking skills and their relevance to health promotion. Int. J. Health Promot. Educ..

[18] Caraher M., Dixon P., Lang T., Carr-Hill R. (1999). The state of cooking in England: The relationship of cooking skills to food choice. Br. Food J..

[19] Golan M., Crow S. (2004). Parents are key players in the prevention and treatment of weight-related problems. Nutr. Rev..

[20] Faith M.S., Scanlon K.S., Birch L.L., Francis L.A., Sherry B. (2004). Parent-child feeding strategies and their relationships to child eating and weight status. Obes. Res..

[21] Spruijt-Metz D., Lindquist C.H., Birch L.L., Fisher J.O., Goran M.I. (2002). Relation between mothers’ child-feeding practices and children’s adiposity. Am. J. Clin. Nutr..

[22] Lang T., Caraher M. (2001). Is there a culinary skills transition? Data and debate from the UK about changes in cooking culture. J. HEIA.

[23] Lavelle F., Hollywood L., Caraher M., McGowan L., Spence M., Surgenor D., McCloat A., Mooney E., Raats M., Dean M. (2017). Increasing intention to cook from basic ingredients: A randomised controlled study. Appetite.

[24] Krueger R.A., Casey M.A. (2014). Focus Groups: A Practical Guide for Applied Research.

[25] McGowan L., Caraher M., Raats M., Lavelle F., Hollywood L., McDowell D., Spence M., McCloat A., Mooney E., Dean M. (2017). Domestic cooking and food skills: A review. Crit. Rev. Food Sci. Nutr..

[26] Lavelle F., McGowan L., Spence M., Caraher M., Raats M.M., Hollywood L., McDowell D., McCloat A., Mooney E., Dean M. (2016). Barriers and facilitators to cooking from ‘scratch’ using basic or raw ingredients: A qualitative interview study. Appetite.

[27] Braun V., Clarke V. (2006). Using thematic analysis in psychology. Qual. Res. Psychol..

[28] Braun V., Clarke V. (2014). What can “thematic analysis” offer health and wellbeing researchers?. Int. J. Qual. Stud. Health Well-Being.

[29] Lavelle F., McGowan L., Hollywood L., Surgenor D., McCloat A., Mooney E., Caraher M., Raats M., Dean M. (2017). The development and validation of measures to assess cooking skills and food skills. Int. J. Behav. Nutr. Phys. Act..

[30] Ronto R., Ball L., Pendergast D., Harris N. (2017). Environmental factors of food literacy in Australian high schools: Views of home economics teachers. Int. J. Consum. Stud..

[31] Lai-Yeung T.W. (2015). Hong Kong parents’ perceptions of the transference of food preparation skills. Int. J. Consum. Stud..

[32] Soliah L.A., Walter J.M., Jones S.A. (2012). Benefits and barriers to healthful eating: What are the consequences of decreased food preparation ability?. Am. J. Lifestyle Med..

[33] Dixon J., Banwell C. (2004). Heading the table: Parenting and the junior consumer. Br. Food J..

[34] Trepka M.J., Murunga V., Cherry S., Huffman F.G., Dixon Z. (2006). Food safety beliefs and barriers to safe food handling among WIC program clients, Miami, Florida. J. Nutr. Educ. Behav..

[35] Jabs J., Devine C.M. (2006). Time scarcity and food choices: An overview. Appetite.

[36] van der Horst K. (2012). Overcoming picky eating. Eating enjoyment as a central aspect of children’s eating behaviors. Appetite.

[37] Russell C.G., Worsley A., Campbell K.J. (2015). Strategies used by parents to influence their children’s food preferences. Appetite.

[38] Allirot X., da Quinta N., Chokupermal K., Urdaneta E. (2016). Involving children in cooking activities: A potential strategy for directing food choices toward novel foods containing vegetables. Appetite.

[39] Kellett M., Forrest R., Dent N., Ward S. (2004). ‘Just teach us the skills please, we’ll do the rest’: Empowering ten-year-olds as active researchers. Child. Soc..

[40] Baumrind D., Brooks-Gunn J., Lerner R., Petersen A.C. (1991). Parenting styles and adolescent development. The Encyclopedia of Adolescence.

[41] Sokol R.L., Qin B., Poti J.M. (2017). Parenting styles and body mass index: A systematic review of prospective studies among children. Obes. Rev..

